# An “Awakener” Patient Suffering From Treatment-Resistant Depression Following Adjunctive Cariprazine

**DOI:** 10.7759/cureus.53246

**Published:** 2024-01-30

**Authors:** Konstantinos Bonotis

**Affiliations:** 1 Department of Psychiatry, University of Thessaly, Larissa, GRC

**Keywords:** add-on treatment, atypical antipsychotics, augmentation agents, cariprazine, d3 receptors, treatment-resistant depression

## Abstract

This report presents a case of particular interest in terms of course and therapeutic outcomes, concerning a patient suffering from treatment-resistant depression in whom adjunctive cariprazine to medication brought about an immediate overall improvement in symptomatology. Informed written consent was obtained from the subject for publication of the case.

## Introduction

Major depression has been recognized as a leading cause of chronic disability worldwide. In addition, treatment-resistant depression represents a hard medical riddle primarily for the patients and their family members as well as for health services [1]. Fortunately, there are various and novel treatment options available, including pharmaceutical and non-pharmaceutical biological therapies. Various compounds have been indicated as add-on therapy to antidepressants along with atypical antipsychotics, mood stabilizers, thyroid hormones, psychostimulants, intranasal esketamine, etc. The clinician’s choices depend on patients’ medical history, comorbidity, and the drug interaction, taking into account the side effects.

Just recently, cariprazine, usually referred to as a third-generation antipsychotic and already being used in the treatment of schizophrenia and bipolar disorder, was approved by the FDA as an add-on treatment in depression, which was hailed as a significant advancement in this field [2]. The argument is based on the particular pharmaceutical properties of cariprazine as well as on a favorable side effect profile [2].

## Case presentation

A 60-year-old man sought a psychiatric evaluation suffering from depressive symptoms that appeared almost two years ago. He presented depressive feelings, anhedonia, loss of energy, psychomotor retardation, lack of spontaneous speech, anorexia, sleep disturbances, decreased libido, avoidance of social contacts, ideas of unworthiness, and transient suicidal ideation. At the same time, his functioning was significantly impaired and he was unable to cope with work demands. No history of depression was mentioned. In the past, almost daily alcohol use was reported, which suddenly stopped on the occasion of a COVID-19 infection when he developed ageusia. Throughout the entire follow-up period to remission, the patient completely abstained from alcohol. The patient has never experienced hypomania/mania before. No family history of psychiatric disorders was mentioned. The patient’s blood tests and brain imaging were negative. No other underlying neurological or systemic diseases were detected. It could be assumed that the preceding alcohol misuse was a risk factor for the appearance of depression in this patient [3].

During the interview, he confirmed the symptomatology without pointing out any associated factors or events. He described that the lockdown period was convenient for him in the sense that he justified his withdrawal from all activities and that his clinophilia was a refuge ("like hiding").

In addition, he complained of persistent tinnitus. According to the patient’s history, tinnitus, as well as other physical complaints (palpitations, chest pain) preceded the depressive symptoms. In particular, tinnitus persisted throughout the episode. Patient Health Questionnaire-9 (PHQ-9) was administered upon evaluation with a score of 22 (PHQ-9 = 22).

By the day of the first visit, he had been taking venlafaxine 75 mg, followed by escitalopram 20 mg daily for a total period of 18 months with no clinical response.

A change of medication was made and combined treatment was administered successively with different antidepressants (vortioxetine, venlafaxine, and clomipramine at the highest recommended doses) as well as adjunctive treatment agents (aripiprazole, lithium, lamotrigine, and quetiapine). In particular, he received vortioxetine 20 mg with aripiprazole 10 mg as an augmenting agent, followed by clomipramine titrated until 300 mg along with aripiprazole 10 mg and lithium 660 mg without response. Meeting the criteria for treatment-resistant depression, he was recommended to undergo electroconvulsive therapy (ECT), which he refused, and repetitive transcranial magnetic stimulation (r-TMS) treatment, which he accepted, but ultimately did not follow. He also refused to be admitted to a psychiatric hospital for the prospect of receiving ketamine. A new treatment modification followed, including a combined daily administration of venlafaxine 375 mg, lamotrigine 100 mg, and quetiapine XR 200 mg. The clinical response was only partial and inadequate. One year later, and while exhibiting good compliance, he showed no substantial improvement. The clinical response was inadequate. This was the medication regimen when cariprazine was added.

In contrast, the addition (without titration) of cariprazine 3 mg brought about a dramatic improvement within a week. He showed progressive remission of almost all symptoms, including tinnitus and primarily loss of energy (PHQ-9 = 8). His motivation was remarkable as he started daily activities that he had abandoned for almost three years. He continued to complain only about reduced libido. The patient did not experience any adverse effects from cariprazine. Ten months later, he is fully recovered. Currently, he receives venlafaxine 150 mg, lamotrigine 100 mg, and cariprazine 3 mg.

## Discussion

The use of cariprazine as an add-on treatment provides an interesting new perspective. It occurred as a result of relevant clinical studies in which the efficacy and safety of cariprazine administration in patients with unipolar depression were demonstrated [4-7]. The recognition of its efficacy in bipolar depression had preceded [8].

The argument that cariprazine is a new specific option [1,2] is also based on its good profile of side effects and interactions with other psychotropic drugs. Compared to other antipsychotics, it is well-tolerated, exhibiting rare side effects such as metabolic syndrome, sedation, and QT prolongation [9]. Most commonly described are dose-related akathisia and restlessness [6,9].

Cariprazine has distinctive pharmaceutical properties. It is a D2/D3 and 5-HT1A partial agonist, has high affinity with partial antagonistic activity for 5-HT2B, and blocks 5-HT2A receptors. Specifically, compared to all other atypical antipsychotics, it possesses a higher binding affinity for D3 receptors than for D2 receptors and, in addition, a much higher binding affinity for D3 receptors than dopamine [10]. A laboratory study showed that chronic administration of cariprazine in rats demonstrated up-regulation of D3 receptor levels in various brain regions, an observation unique among antipsychotics [11]. D3 receptors are detected more favorably in areas of the brain of the limbic system related to the regulation of reward-related behavior, emotion, and motivation [4]. It could be therefore hypothesized that the reported specificity of action is potentially related to its efficacy in the treatment of depressive symptoms by enhancing patient motivation [12,13].

Large sample, multicenter, updating studies comparing efficacy and tolerance of different atypical antipsychotics as augmentation agents could be more enlightening [14-16].

## Conclusions

The referred patient presented an episode of major depression. This episode lasted for almost three years as he did not respond to the administration of antidepressants of different classes in sufficient doses and time periods, as well as to the combined administration of augmentation agents, meeting the criteria of treatment-resistant depression. In contrast, he responded immediately to cariprazine administration showing almost complete remission of symptoms as well as stabilization. His mobilization, with concomitant remission of core symptoms such as loss of energy and anhedonia, was remarkable. A review of the existing literature suggests that this observation might not be an incidental finding.

Further research in this field should be evaluated as a scientific challenge taking into account clinical relevance. Studies in subpopulations of patients with a dual diagnosis or history of alcohol misuse where the reward system is involved in the pathophysiology, given the described role of D3 receptors, may be of particular interest. Currently, cariprazine already represents a valuable choice in clinical reasoning, where generic planning according to guidelines should be complementary to a personalized approach for each patient.
