# Correction: An “Awakener” Patient Suffering From Treatment-Resistant Depression Following Adjunctive Cariprazine

**DOI:** 10.7759/cureus.c363

**Published:** 2025-10-15

**Authors:** Konstantinos Bonotis

**Affiliations:** 1 Department of Psychiatry, University of Thessaly, Larissa, GRC

This article has been corrected to disclose a financial relationship between the author, Konstantinos Bonotis, and Recordati, a pharmaceutical company which manufactures cariprazine. Konstantinos Bonotis served as an advisory board member for Recordati in December, 2022. As a result, an honorarium was awarded to the University of Thessaly's Research Committee. 

